# Multilayer optical thin film design with deep Q learning

**DOI:** 10.1038/s41598-020-69754-w

**Published:** 2020-07-29

**Authors:** Anqing Jiang, Yoshie Osamu, Liangyao Chen

**Affiliations:** 1 Graduate School of IPS, Waseda University, Fukuoka, Japan; 2 Department of Optical Science and Engineering, Fudan University, Shanghai, China

**Keywords:** Metamaterials, Computer science

## Abstract

Multilayer optical film plays a significant role in broad fields of optical application. Due to the nonlinear relationship between the dispersion characteristics of optical materials and the actual performance parameters of optical thin films, it is challenging to optimize optical thin film structure with the traditional models. In this paper, we present an implementation of Deep Q-learning, which suited for the most part for optical thin film. As a set of concrete demonstrations, we optimize solar absorber. The optimal program could optimal this solar absorber in 500 epoch (about 200 steps per-epoch) without any human intervention. Search results perform better than researchers’ manual searches.

## Introduction

Since 1970, with the development and application of computer technology, a variety of numerical data processing methods emerge in endlessly. Many numerical optimization algorithms, such as linear programming, simplex solution searching, least-square or damped-least-square error reductions, were studied and applied for the optical thin film design. Which the least-square error reduction was one of the most successful methods in the application. Those traditional methods often show the limitation of focusing only on the solution searching for the film system’s local optimum with a complicated multilayer film structure. The optical film researchers also developed a variety of global optimization methods used in the design of the film structure. Tikhonravov and Trubetskov developed an optical coating design software based on the needle optimization technique^1^. Sullivan and Dobrowolski implemented the needle optimization method to improve it much more flexible by defining a merit function that consists of quite complicated spectral quantities^2^. Chang and Lee applied the generalized simulated-annealing method (GSAM) for the thin-film system design and discovered that there would be no problem with the trapping local minimum that happened in the design^3^. Recently, the researchers worked in this field also have applied to the optical coating optimization methods with several models such as particle swarm optimization (PSO)^4^, genetic algorithm (GA)^5,6^, ant colony algorithm^7^ and deep learning algorithm^8–12^.

In the traditional methods, as mentioned above, most of the optimization algorithms are based on the assumption that the film materials are not optically dispersive. i.e., the traditional algorithms only consider the parameters related to the refractive index and thickness of materials. With the increase of the spectral bandwidth, the dispersion feature of the material will significantly affect the film’s optical properties. Because of the broad bandwidth, the refractive indices of all materials vary as a function of wavelength. Extinction coefficients of the materials should be considered in some conditions, like that of the films in solar absorption applications. With the increase of the parameters that need to be optimized, the above classical algorithms’ optimization performance will significantly reduce or even be less able to work usually. An optimization algorithm framework with strong search ability and better robustness need to be used in the field of film optimization. Since 2014, with the rapid development of computer science, especially artificial intelligence, deep learning was widely applied in many research fields. Deep learning has been pioneeringly applied in many physical problems recently^13–15^. Although the above research has solved the related physical problems, it needs a lot of known data. This supervised learning model can not solve the problem well for some unknown combination problems of materials and structures. Especially in the optimization problem, because the search space is extensive, supervised learning can not adequately predict and optimize the optical thin film structure. The total optical film optimization method can be regarded as Markov Decision Process (MDP), which can be solved by reinforcement learning (RL). As one of the important reinforcement learning branches, deep Q-learning (DQN) has achieved a lot of success in the field of optimization and control and has made significant advantages in the confrontation with human beings^16,17^.

In this work, a new multi-layered film optimization method is proposed with the DQN. The algorithm will automatically search the thickness of each layer of the film that minimize the objective function. Each layer of the film materials will be selected from a given set of pre-selected materials. In the following sections, we show the details of the algorithm’s formulation, and as a demonstration, we apply the deep Q-learning algorithm to the optimization for both a solar absorption film and an anti-reflection film structure. In the following sections, this paper will introduce in detail how to combine DQN with optical multilayer film optimization and show the performance of the algorithm in several typical film systems.

## Optimization target

For optical thin films, three main interrelated factors determine the optical properties of the film, respectively, transmission (T), reflection (R), and absorption (A). The relationship between transmission, reflection and absorption is determined by the conservation of energy, i.e.

1 $$\begin{aligned} R + T + A = 1 \end{aligned}$$

For the photon energy conservation of the light entering into the material and structure, they are implying that the ideal solar absorber will correspond to the feature of that $T = 0$, $R = 0$, and $A = 1$. For the anti-reflective film, the feature is aiming at T = 1, R = 0, and A = 0. The filter film has different requirements for the characteristics at different wavelengths range. The transmission is 0 to cut off incident light in a specific wavelength range, and the transmission is 1 in other wavelengths.

For multi-layered optical thin film optimization, especially for broadband optimization,the target optical properties of the thin film are the optical performance constants of the material, as the reflective indices ${\varvec{n}}(\lambda ) = [n_1(\lambda ), \ldots , n_K(\lambda )]$ and extinction coefficient ${\varvec{k}}(\lambda ) = [k_1(\lambda ), \ldots , k_K(\lambda )]$, as well as the thickness ${\varvec{d}}=[d_1,\ldots ,d_K]$ of the *K* layers material. In addition, in order to design multi-layer structure, we express the target spectrum which contains transmission, reflection and absorption as $S^*(\lambda , \theta )=[A^*(\lambda , \theta ), T^*(\lambda , \theta ), R^*(\lambda , \theta )]$, where $\theta$ is the incident angle and $\lambda$ is the wavelength. To search the best optimum thin film structure, we aim to minimize the residual between the spectrum of the given structure and the target spectrum. The absolute error merit function $AE({\varvec{n}},{\varvec{k}},{\varvec{d}})$ is defined as:

2 $$\begin{aligned} AE({\varvec{n}}, {\varvec{k}}, {\varvec{d}}) = \sum _{\lambda ,\theta }W(\lambda )\left| S(\theta , \lambda ; {\varvec{n}}, {\varvec{k}}, {\varvec{d}}) - S^*(\lambda , \theta )\right| \end{aligned}$$

where $W(\lambda )$ describes the weight associated with each wavelength, in the optimization task of this method, the thin film uses artificially selected materials for each layer. It means giving the desired spectrum $S*(,)$, the optimization problem can be formulated as the following:

3 $$\begin{aligned} {\varvec{d}}^* = \mathop {argmin}_{{\varvec{d}}\in {\mathbb {R}}^K}\ AE({\varvec{d}}) \end{aligned}$$

where $d^*$ represent the optimal layer thickness. In general, it is NP-hard to find the exact global optimum of discrete optimization. This optimization problem is only exponential and solvable. Having defined the optimization problem by (3), we now outline the use of deep Q-learning in finding the optimal multi-layered structure. In particular, the film simulation environment will be used as the *environment* of reinforcement learning, and the improvement of multilayer film performance will be used as a condition of defining *return*. As for the *actions* of DQN agent will adjust the film thickness.

## Implementation of deep Q-learning

**Figure 1:**
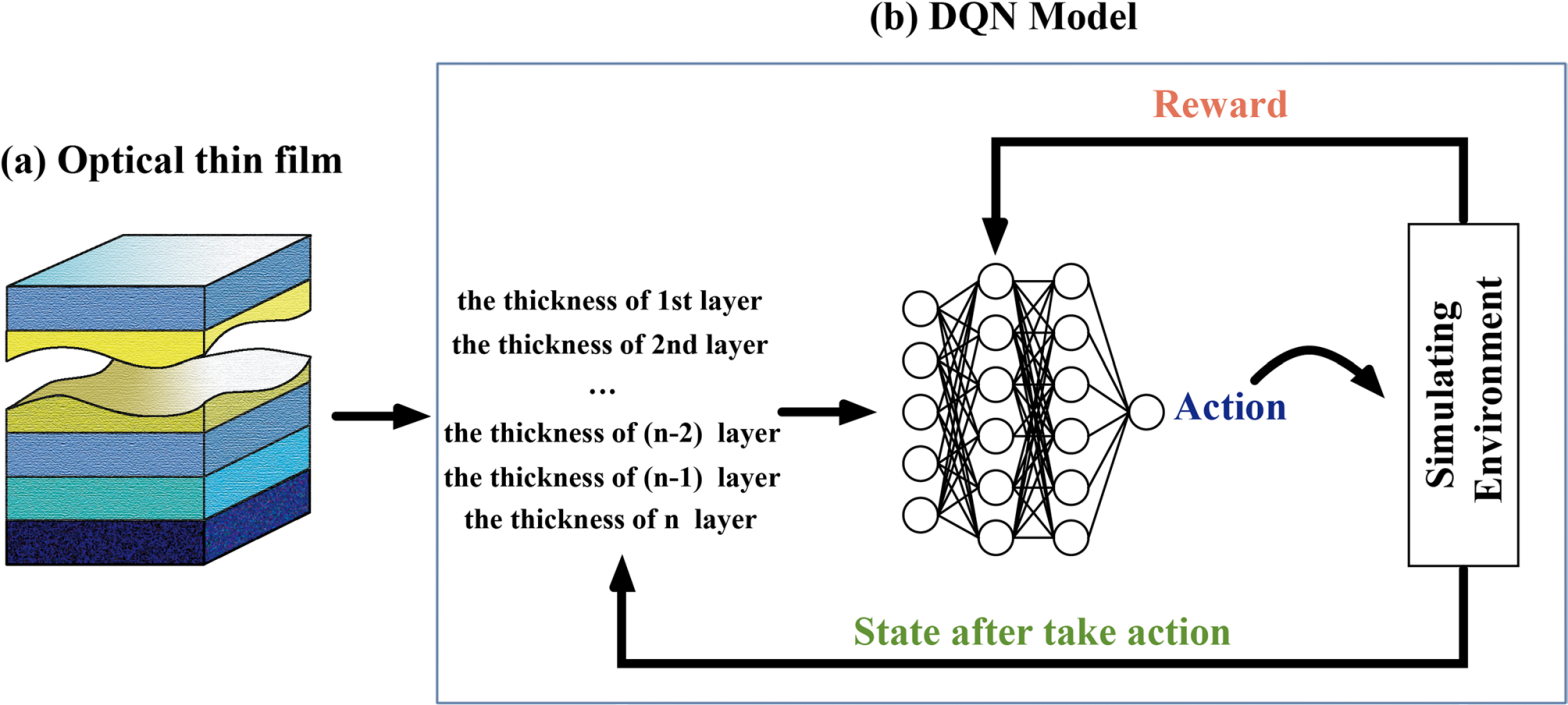
(**a**) Multilayer optical thin film structure. (**b**) The deep Q-learning system used for finding the best optical thin film structures which can meet optimization target.

Here we explain the method briefly and discuss the implemention of deep Q-learning to solve the multilayer optical film problem. We use DQN to iteratively optimize the film parameters in the simulation environment, and get a better simulation film structure after a certain number of rounds of optimization. Figure 1 shows the optimization process of the optical thin film.

### Environment

The environment of DQN is based on one kind of multilayer optical film simulation algorithm called the transfer matrix method (TMM)^18^. The efficiency of TMM is higher than in another optical film environment FDTD. In this environment, the film thickness ${\varvec{d}}$ can be modified by actions from the policy. The environment can simulate the film’s optical performance: *A*, *T*, and *R*.

### States

In this reinforcement learning, we input two kinds of information as states, respectively the optical properties of the film and the thickness of the film. The film’s optical properties represents the absorption, transmittance, and reflectivity in the target wavelength range. For example, because the optical parameters at each wavelength are different, the input shape of this part states is $3\ *$ wavelength $*$ wavelength resolution. The second part of the state is the thickness of ${\varvec{d}}$ of each layer. For the thickness state, the thickness of the film is limited to the range of 1–300 nm. When the state exceeds this range, the model receives a negative reward and start a new round of optimization.

### Actions

Actions represent the operation at each step in the process of optimizing the film structure. These actions adjust film thickness at different scales. As for a film with four layers of film thickness that need to be optimized, the minimum optimization scale is 0.1 nm. The actions space contains $4 \times 6 = 24$ different actions. The list of all the actions is shown in Table 1.

**Table 1:** Definition of actions used in DQN.

Action no.	Action definition
0	Decrease the 1st layer by 10 nm. (min 1 nm)
1	Decrease the 1st layer by 1 nm. (min 1 nm)
2	Decrease the 1st layer by 0.1 nm. (min 1 nm)
3	Increase the 1st layer by 10 nm. (max 500 nm)
4	Increase the 1st layer by 1 nm. (max 500 nm)
5	Increase the 1st layer by 0.1 nm. (max 500 nm)
6	Decrease the 2nd layer by 10 nm. (min 1 nm)
7	Decrease the 2nd layer by 1 nm. (min 1 nm)
8	Decrease the 2nd layer by 0.1 nm. (min 1 nm)
9	Increase the 2nd layer by 10 nm. (max 500 nm)
10	Increase the 2nd layer by 1 nm. (max 500 nm)
11	Increase the 2nd layer by 0.1 nm. (max 500 nm)
12	Decrease the 3rd layer by 10 nm. (min 1 nm)
13	Decrease the 3rd layer by 1 nm. (min 1 nm)
14	Decrease the 3rd layer by 0.1 nm. (min 1 nm)
15	Increase the 3rd layer by 10 nm. (max 500 nm)
16	Increase the 3rd layer by 1 nm. (max 500 nm)
17	Increase the 3rd layer by 0.1 nm. (max 500 nm)
18	Decrease the 4th layer by 10 nm. (min 1 nm)
19	Decrease the 4th layer by 1 nm. (min 1 nm)
20	Decrease the 4th layer by 0.1 nm. (min 1 nm)
21	Increase the 4th layer by 10 nm. (max 500 nm)
22	Increase the 4th layer by 1 nm. (max 500 nm)
23	Increase the 4th layer by 0.1 nm. (max 500 nm)

For more film layers, the actions space can be expressed by as follows.

4 $$\begin{aligned} Actions =\{action|\pm action=10^{-K}, 0\ge K\ge N, K\in Z^{+}\} \end{aligned}$$

### Q-network

**Figure 2:**
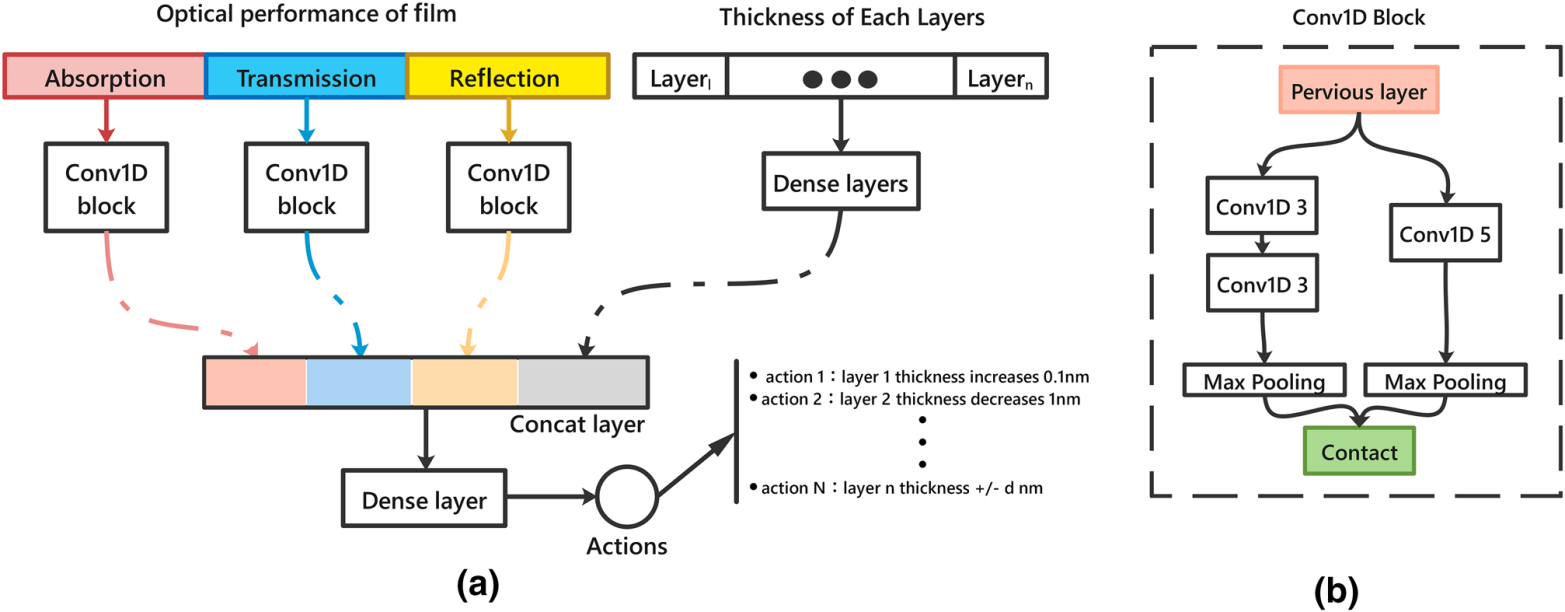
(**a**) A Q-network with two parts of information is introduced. (**b**) One-dimensional convolution network block (Conv1D Block) is used to extract the performance features of optical thin films.

When transplanting DQN, we use a multi-input neural network instead of a full connection neural network as the backbone network of DQN. According to Fig. 2, the input of the network is divided into two parts, which represent the information of film performance states and thickness states, respectively. As for film performance, we implement a conv1D block to extract different optical performance. Each Conv1D block consists of 2 different convolution kernels, which kernels size is 3 and 5, respectively. To keep the output consistency of the Conv1D block and to reduce the number of network parameters, a maximum pooling layer is taken as the last layer of this block. The state’s network consists of three layers Fully connected neural network. In the last part of the DQN, the two subnetworks are combined to build a complete Q-network. The spliced information is transmitted to the final actions through a dense layer of 128 hidden units. We use relu function as activation function of Q-network.

### Rewards

The reward is essential feedback used to measure the performance of the agent in reinforcement learning. It represents the next gain in a single time step after acting *A* in the state *S*. In our application, shows in Table 2, this reward value *R* indicates whether the modification of the film structure is helpful to optimize the film parameters. Considering 1 (satisfying the optimization goal) and − 1 (not satisfying the optimization goal) is the return value, it will not be easy to guide the agent to find the appropriate optimization direction. We have designed returns value in two patterns, binary and continuous, for reward shaping. The reward function is designed with the idea of reward shaping, to avoid sparse reward problem.

**Table 2:** Definitions of actions used in DQN.

Reward	Reward value
Film thickness out of limit	− 1
Film performance not been improved in threshold step	− 1
Film performance not been improved	− 0.01
Film performance been improved	Observation loss
Film performance meet target	1

which observation loss is represent improvement in last step.

### Exploration and exploitation

At first, the Q network is initialized randomly, and a series of predictions are also random. If we choose the action with the highest Q value, the action is naturally random. At this time, the agent is doing “exploration”. As the Q function converges, the returned Q value will tend to be the same. It allows the model to converge faster, and also convergence being wrongly affected in the local optimal search process. It can increase the convergence speed of the model, but it will also cause the model to fall into a local trap. A simple and effective solution is $\epsilon -greedy$ exploration, which uses probability $\epsilon$ to choose whether to continue exploration or to make decisions directly based on experience.

### DQN algorithm training rule

Combining the methods as mentioned above, the Q-network can be optimized by the algorithms as below:
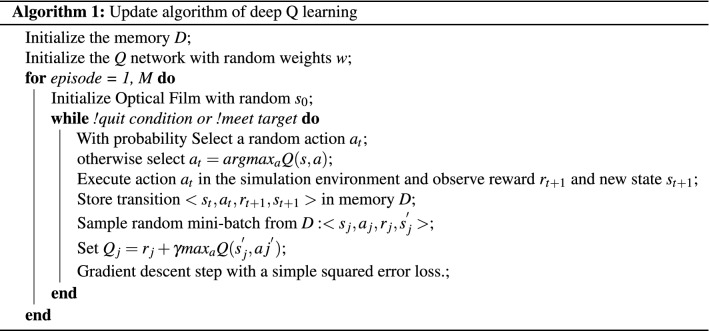
 where ${\varvec{D}}$ is the memory pool that stores the experienced data, extract a part of the data from memory for updating each time when updating parameters, to break the association between data. ${\varvec{M}}$ is the total number of times of reinforcement learning.

## Experiment

### Solar selective absorber

Solar selective absorber plays an essential role in the field of solar energy utilization. For the film, a full absorption bandwidth and higher absorption efficiency will be required to improve the film’s performance. Optimizing the film structure becomes very important, but the optimization method using a genetic algorithm or simplex method usually needs an excellent initial film structure in practice. We use the DQN algorithm to optimize the selective solar absorber to verify the effectiveness of the algorithm. We use materials optical constant from^1,19^.

First, we define the optimized objective function. The spectral range of solar radiation is extensive, while energy is mainly concentrated in the visible and near-infrared spectrum ($250{-}2500$ nm). Based on Stefan-Boltzmann’s law, the total emissive power of a blackbody is proportional to the fourth power of its temperature, which is a placement law that shows that blackbody radiation exists the peak values at different temperatures. As the temperature increases, the maxima radiation increases, and the corresponding wavelength decreases.

**Figure 3:**
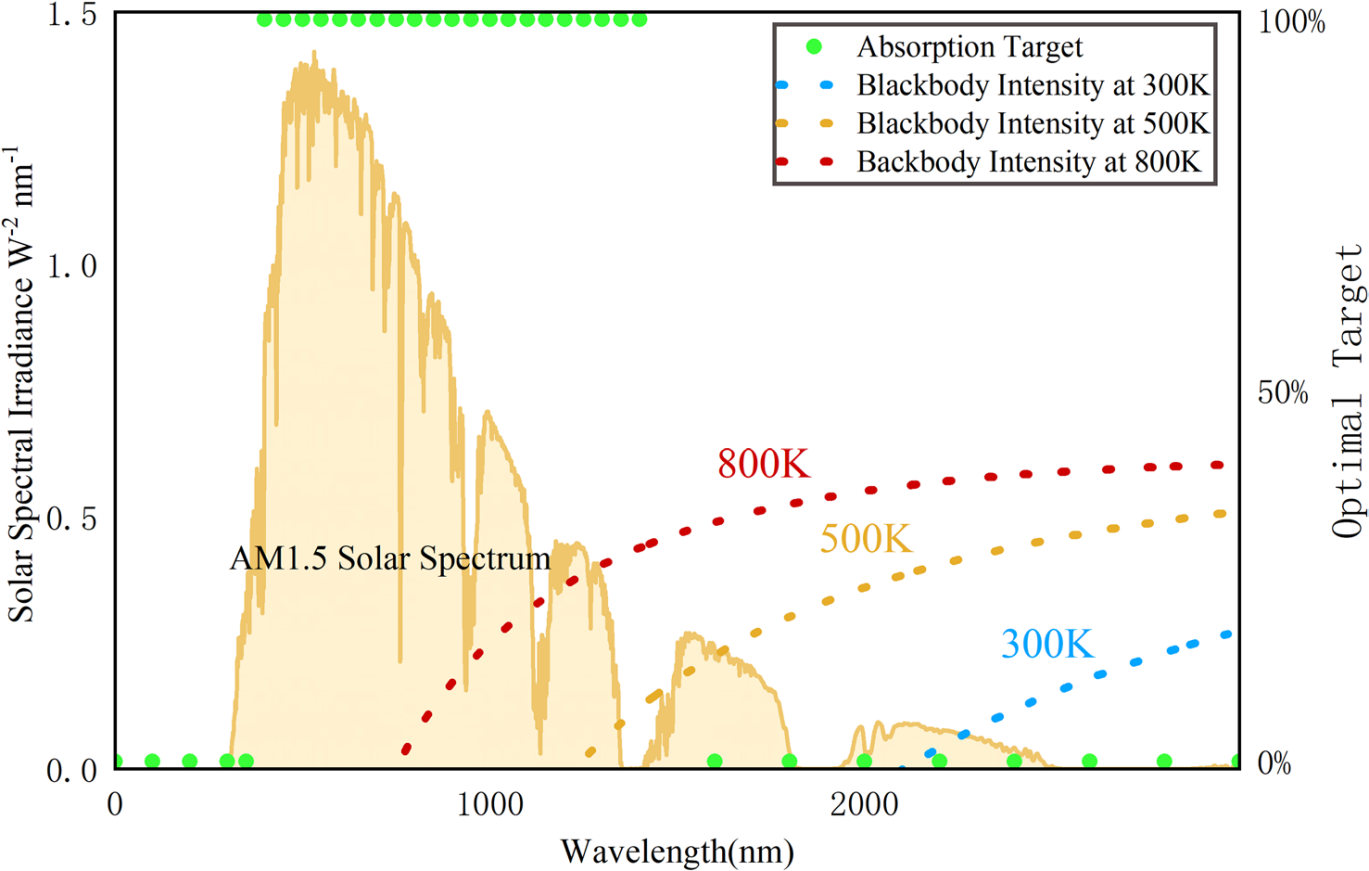
Plot of the optimal target, solar spectrum and blackbody radiation.

Here, we employ the deep Q-learning to optimize the performance of the solar energy selective absorption of the film. As shown in Fig. 3, the primary power of solar energy is mainly concentrated in the wavelength range of 300–1400 nm.

Thus we design a multilayered film structure to have higher solar energy absorption efficiency in the 300–1400 nm wavelength range. The layered materials consisting of the film structure are Ti and SiO2, respectively. The 200-nm-thick Cu layer is used as the substructure to ensure that the film device is opaque to have zero transmission of the light in the entire working wavelength range. The thickness of each layer is initialized with a random value. We use the deep Q-learning in sequence to optimize the thickness of each layer of the film structure. To verify the effectiveness of the algorithm, we optimized three types of the film structures with different layer numbers: (1) 4 layers; (2) 6 layers; and (3) 8 layers. The optimal thickness of each layer is shown in Table 3. The optimizer obtains the best results for each of the three structures, respectively 87.4%, 90.15% and 94.55%. More layers in one film can search for better optimization results.

**Table 3:** Material composition and thicknesses for the optimized solar selective absorber using 2 materials.

Layer#	Material	4 layers (nm)	6 layers (nm)	8 layers (nm)
0	Air	–	–	–
1	SiO$_2$	132.4	126.0	63.19
2	Ti	13.74	6.46	3.47
3	SiO$_2$	77.5	73.37	71.46
4	Ti	–	12.98	6.19
5	SiO$_2$	–	54.56	65.84
6	Ti	–	–	12.45
7	SiO$_2$	–	–	52.4
Sub	Cu	200	200	200
Absorption (%)		87.4	90.15	94.55

**Figure 4:**
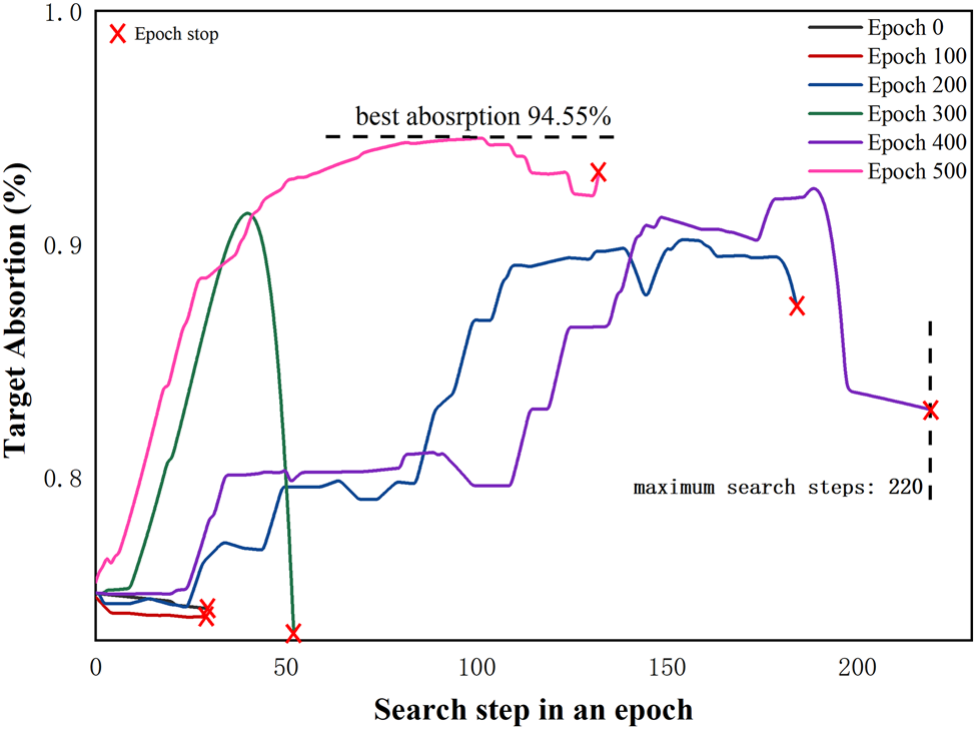
8 layers solar absorption film’s optimization result in different epoch.

Figure 4 expresses the performance of the DQN agent in the search process and optimization. We found that in the first 100 attempts, the agent almost stopped searching very quickly. In the 200th attempt, the agent got a relatively good optimization result. In the 300th attempt, the agent experienced some overfitting, and the optimization score quickly increased and then decreased rapidly. This situation is because the agent repeatedly executes the same action that obtains a good score. Compared with epoch-400 and epoch-500, it can be seen that the training score and the optimal step size to achieve better results are obviously shortened.

## Conclusions

We propose a novel algorithm to optimize the thickness of selective solar absorber, and this reinforcement learning method is easy to extend to other multi-layers films. This algorithm dramatically improves the optimization speed of the film thickness, and the researchers have not given any initialization of the film structure. The network structure using the 1D-Conv network, like backbones, features the observation from the simulation environment. We also notice that the total time-consuming optimization is very controllable, and the total number of iterations can search for a satisfactory result in 500 epochs as we test the result in a PC with AMD 3600X CPU and NVIDIA GTX-1080Ti GPU. The overall optimization time cost is about 20 min. Since the overall operation time is mainly concentrated in the system simulation stage, the overall optimization time may be further shortened, considering the introduction of multi-process simulation.
